# Association analysis between SNPs in *LATS1* and *LATS2* and non-cardia gastric cancer

**DOI:** 10.1186/s12876-020-01250-x

**Published:** 2020-05-18

**Authors:** Li-cong Ma, Xu-yang Tian, Fang Gao, Wen-jie Dong, Tong Dang, Yan-bin Jia

**Affiliations:** 1 Inner Mongolia Institute of Digestive Diseases, The Second Affiliated Hospital of Baotou Medical College, 30 Hudemulin Street, Baotou, 014030 Inner Mongolia China; 2 Pathology Department, the Second Affiliated Hospital of Baotou Medical College, Baotou, 014030 Inner Mongolia China; 3 School of Basic Medicine and Forensic Medicine, Baotou Medical College, Baotou, 014060 Inner Mongolia China; 4 School of Medical Technology, Baotou Medical College, Baotou, 014060 Inner Mongolia China; 5 School of Nursing, Baotou Medical College, 31 Jianshe Road, Baotou, 014060 Inner Mongolia China

**Keywords:** LATS1, LATS2, Non-cardia gastric cancer, Association study

## Abstract

**Background:**

Many studies have found that large tumor suppressor kinase 1 (LATS1) and LATS2 play important roles in many diseases, but studies have been rare on the relationship between these genes and non-cardia gastric cancer (GC). We performed a case-control association study to investigate the associations between single nucleotide polymorphisms (SNPs) in *LATS1* and *LATS2* genes and *Helicobacter pylori* (*H. pylori*) infection as well as the risk of non-cardia GC.

**Methods:**

First, *H. pylori* infection was determined by the serological test using enzyme-linked immunoassay. Then genotyping of SNPs was performed for 808 samples by the Taqman method. Finally, unconditional logistic regression was used to calculate the odds ratios (ORs) and 95% confidence intervals (CIs), adjusted for age and gender, for the association of each SNP with the infection of *H. pylori*, the risk of non-cardia gastric cancer, as well as the expression of LATS1 and LATS2 proteins in non-cardia GC tissues, using the codominant, dominant, recessive, overdominant, and log-additive inheritance models, respectively.

**Results:**

The statistical results showed that *LATS2* rs9552315 was associated with *H. pylori* infection, and the CC + CT genotype could reduce the risk of *H. pylori* infection (odds ratio [OR]: 0.549, 95% confidence interval [CI]: 0.339–0.881, *P* < 0.05) compared with the TT genotype in a dominant model. *LATS1* rs9393175 was associated with the risk of non-cardia GC, and the AG genotype reduced the risk of non-cardia GC (OR: 0.702, 95% CI: 0.516–0.952, *P* < 0.05) compared with the GG + AA genotype in an overdominant model. *LATS2* rs9509492 was associated with the risk of GC in an log-additive model. No associations were found between five SNPs and expression of LATS1 and LATS2 proteins in non-cardia GC tissue.

**Conclusions:**

*LATS2* rs9552315 CT genotype may be a protective factor against infection of *H. pylori*. *LATS1* rs9393175 AG genotype and *LATS2* rs9509492 GG genotype may be protective factors for non-cardia GC.

## Background

Gastric cancer (GC) is one of the most common malignant tumors with high morbidity, high mortality, and poor prognosis. In 2018, there were more than 1 million new cases of GC worldwide and 783,000 deaths caused by GC. The morbidity and mortality rate ranked fifth and third among all malignant tumors, respectively, and it was one of the malignant tumors that seriously affected human health [1]. *Helicobacter pylori* (*H. pylori*) infection is a main cause of GC. According to the epidemiological investigation, about 78% of GC patients have been infected with *H. pylori* [2]. It is closely related to the pathogenesis of GC and was defined as a class I carcinogen of the stomach by the International Agency for Research on Cancer in 1994 [3]. It has been reported in the literature that about 74.7–89.0% GC is associated with *H. pylori* [4]. However, although about 50% of the world’s population is infected with *H. pylori*, only 1–3% eventually develop GC. This suggests that individual genetic factors of the host play important roles in the development of GC [5, 6]. Many literature reports and previous studies from our research group found that the single nucleotide polymorphisms (SNPs) of some genes are associated with *H. pylori* infection and GC [7].

Large tumor suppressor kinase 1 (LATS1) and LATS2 are tumor suppressor proteins that have been discovered in recent years [8]. They are the core components of the Hippo signaling pathway and regulate a series of biological behaviors through phosphorylation reactions with components upstream and downstream of the pathways including cell proliferation, differentiation, apoptosis, and regulation of organ size [9–11]. Studies have reported that the expression levels of LATS1 and LATS2 proteins in malignant tumor tissues such as ovarian tumors and non-small-cell lung carcinoma are significantly lower than those in normal tissues [12, 13]. Previous studies from our group confirmed that the expression levels of LATS1 and LATS2 proteins in GC tissues are significantly lower than those in normal gastric tissues [14]. Therefore, we speculated that *LATS1* and *LATS2* gene polymorphisms may be involved in the occurrence and development of GC. However, the association of *LATS1* and *LATS2* gene polymorphisms with non-cardiac GC carcinogenesis has not been reported.

Therefore, this case-control association study was performed in the Baotou Han population to investigate the association between five SNPs of *LATS1* and *LATS2* genes and *H. pylori* infection and non-cardia GC.

## Methods

### Subjects

We collected 381 blood samples from non-cardia GC patients from Baotou Cancer Hospital (Inner Mongolia, China) from 2008 to 2017, of which 288 cases were collected from June 2008 to December 2010, and the remaining 93 cases were collected from 2015 to 2017. During the same period the patients were recruited, our group collected 427 blood samples from normal medical examinations in the First Affiliated Hospital of Baotou Medical College and Inner Mongolia Baotou Steel Hospital. The inclusion criteria for the cases were as follows. The patients had to be of Han descent, and the three generations had no history of intermarriage with other ethnic groups and had been living in Baotou for more than 5 years at the time of onset. Secondary cases, relapse cases, and patients who had received chemotherapy or radiotherapy were excluded. All cases were confirmed by pathology examination. The normal control inclusion criteria were as follows. The medical examination was required to confirm Han descent, and the three generations had no history of marriage with other ethnic groups and had been living in Baotou for more than 5 years with no individual history of cancer or identifiable gastric or genetic disease. At recruitment, informed consent was obtained from each subject, and the study was approved by the Institutional Review Board of Baotou Medical College.

### *H. pylori* infection test

The serological status of *H. pylori* infection was determined by an enzyme-linked immunosorbent assay (ELISA) using the “Human Helicobacter pylori antibody (HP-Ab) ELISA Kit (96-well plate),” which was purchased from Suzhou Ailsa Bio-Technology Co., Ltd. (Suzhou, China).

### Screening for SNPs

SNPs of the *LATS1* and *LATS2* genes were screened at the National Center for Biotechnology Information database, and the tag SNPs of the study genes were screened according to the Chinese Han genetic polymorphism data provided by the HapMap database (http://hapmap.ncbi.nlm.nih.gov/). The SNP minority allele frequency was required to be greater than 5%, and the linkage disequilibrium (LD) (r^2^) cut-off was required to be 0.8. A total of five SNPs were screened including *LATS1* rs9393175, *LATS2* rs558614, rs9552315, rs7317471, and rs9509492.

### Genotyping assay

Genomic DNA was extracted using the “TIANamp Blood DNA Kit, spin-column type (200 tests)”, which was purchased from Tiangen Biotech (Beijing) Co., Ltd. Genotyping was performed by the TaqMan method. Primers and probes were designed by ABI Scientific Inc. (Sterling, VA, USA). The genotyping success rates of five SNPs were all more than 97.8%. Failed genotypes were not repeated. In the experiment, 5% of DNA samples with good quality and quantity were randomly selected for repeated experiments to verify the accuracy of the results. The consistency of genotyping results in all repeated samples was 100%.

### Statistical analysis

R software (version 3.5.0) was used for statistical analysis. Age did not conform to the normal distribution, so the nonparametric rank-sum test was adopted to test the difference between cases and controls. Categorical data such as gender were evaluated using the chi-square test. The Pearson’s chi-square test of the genetics package with R software was used for Hardy Weinberg Equilibrium (HWE) in the case and control groups. Unconditional logistic regression was used to calculate the odds ratios (ORs) and 95% confidence intervals (CIs), adjusted for age and gender, for the association of each SNP with the infection of *H. pylori*, the risk of non-cardia gastric cancer, as well as the expression of LATS1 and LATS2 proteins in non-cardia GC tissues, using the codominant, dominant, recessive, overdominant, and log-additive inheritance models, respectively. Haploview 4.2 software was used to conduct LD and haplotype block on the four SNPs of LATS2, calculate the limit of detection value and linkage disequilibrium, and construct haplotype by the D’ confidence interval method.

## Results

### General description of the samples

A total of 808 samples were included in this study, including 381 samples of non-cardia GC (case group) and 427 samples of healthy individuals (control group). The statistical results showed that the age distribution between the case group and the control group was statistical significance. There was no significant difference in gender distribution between the case group and the control group (Table 1).

**Table 1:** Comparison of general conditions of 808 samples

Variable		Group		Statistics	*P*
		Case (*n* = 381)	Control (*n* = 427)		
Age (median (IQR))		60 (52–69)	57 (50–65)	W = 65,012	< 0.001
Age group	< 65	237 (62.2%)	313 (73.3%)	χ2 = 11.408	< 0.001
	≥65	144 (37.8%)	114 (26.7%)		
Gender	female	95 (24.9%)	107 (25.1%)	χ2 = 0.002	0.968
	male	286 (75.1%)	320 (74.9%)		

### Association between *LATS1* and *LATS2* SNPs and risk of *H. pylori* infection in normal control

Among the 427 normal subjects, 225 cases were negative for *H. pylori* infection and 202 were positive for *H. pylori* infection, with a positive rate of 47.3%. The statistical results showed that there were no significant differences in the age and gender distribution between the *H. pylori*-negative and *H. pylori*-positive infection groups (Table 2). The genotype distribution of all SNPs was consistent with HWE in the control group. The results showed that *LATS2* rs9552315 was associated with infection of *H. pylori* in codominant and dominant models. The dominant genetic model was more suitable with a lower Akaike Information Criterion (AIC) and Bayesian Information Criterion (BIC). No association was found between other SNPs and infection of *H. pylori* (Table 3). Furthermore, no haploid blocks were formed between the four SNPs of *LATS2*.

**Table 2:** General comparison in negative and positive cases of *H. pylori* infection

Variable		*H. pylori* infection of the control group		Statistics	*P*
		negative (*n* = 225)	positive (*n* = 202)		
Age (median (IQR))		54 (50–66)	53 (49–64)	W = 24,220	0.240
Age group	< 65	160 (71.1%)	153 (75.7%)	χ2 = 1.167	0.280
	≥65	65 (28.9%)	49 (24.3%)		
Gender	female	56 (24.9%)	51 (25.2%)	χ2 = 0.007	0.932
	male	169 (75.1%)	151 (74.8%)

**Table 3:** Association between 5 SNPs and risk of *H. pylori* infection under different genetic models

Locus	Model	Genotype	Control [n(%)]	Case [n(%)]	OR (95%CI)^a^	*P*	AIC	BIC
LATS1 rs9393175	codominant	G/G	125 (55.56)	128 (64.00)	1	–	1092.330	1115.760
		A/G	88 (39.11)	61 (30.50)	0.671 (0.443–1.010)	0.057		
		A/A	12 (5.33)	11 (5.50)	0.916 (0.383–2.175)	0.842		
	dominant	G/G	125 (55.56)	128 (64.00)	1	–	591.691	607.899
		A/A + A/G	100 (44.44)	72 (36.00)	0.700 (0.472–1.035)	0.074		
	recessive	G/G + A/G	213 (94.67)	189 (94.50)	1	–	594.873	611.081
		A/A	12 (5.33)	11 (5.50)	1.062 (0.449–2.487)	0.889		
	overdominant	G/G + A/A	137 (60.89)	139 (69.50)		–	591.254	607.462
		A/G	88 (39.11)	61 (30.50)	0.675 (0.449–1.011)	0.058		
	log-additive	–	–	–	0.792 (0.570–1.093)	0.158	592.876	609.084
LATS2 rs558614	codominant	T/T	63 (29.30)	60 (30.46)	1	–	579.405	599.511
		C/T	106 (49.30)	91 (46.19)	0.900 (0.572–1.416)	0.648		
		C/C	46 (21.40)	46 (23.35)	1.042 (0.606–1.794)	0.881		
	dominant	T/T	63 (29.30)	60 (30.46)	1	–	577.742	593.826
		C/C + C/T	152 (70.70)	137 (69.54)	0.943 (0.617–1.442)	0.787		
	recessive	T/T + C/T	169 (78.60)	151 (76.65)	1	–	577.614	593.698
		C/C	46 (21.40)	46 (23.35)	1.112 (0.698–1.772)	0.654		
	overdominant	T/T + C/C	109 (50.70)	106 (53.81)	1	–	577.428	593.512
		C/T	106 (49.30)	91 (46.19)	0.884 (0.599–1.303)	0.534		
	log-additive	–	–	–	1.012 (0.773–1.326)	0.930	577.807	593.891
LATS2 rs9552315	codominant	T/T	45 (20.18)	60 (30.00)	1	–	588.160	608.397
		C/T	119 (53.36)	88 (44.00)	0.549 (0.339–0.881)	0.014		
		C/C	59 (26.46)	52 (26.00)	0.650 (0.377–1.114)	0.118		
	dominant	T/T	45 (20.18)	60 (30.00)	1	–	586.672	602.862
		C/C + C/T	178 (79.82)	140 (70.00)	0.582 (0.370–0.909)	0.018		
	recessive	T/T + C/T	164 (73.54)	148 (74.00)	1	–	592.331	608.521
		C/C	59 (26.46)	52 (26.00)	0.970 (0.626–1.499)	0.891		
	overdominant	T/T + C/C	104 (46.64)	112 (56.00)	1	–	588.616	604.805
		C/T	119 (53.36)	88 (44.00)	0.685 (0.466–1.005)	0.054		
	log-additive	–	–	–	0.811 (0.618–1.061)	0.128	590.018	606.207
LATS2 rs7317471	codominant	C/C	177 (80.09)	173 (85.64)	1	–	590.953	611.190
		C/T	39 (17.65)	28 (13.86)	0.746 (0.435–1.266)	0.281		
		T/T	5 (2.26)	1 (0.50)	0.206 (0.011–1.230)	0.152		
	dominant	C/C	177 (80.09)	173 (85.64)	1	–	590.590	606.780
		C/T + T/T	44 (19.91)	29 (14.36)	0.684 (0.405–1.143)	0.151		
	recessive	C/C + C/T	216 (97.74)	201 (99.50)	1	–	590.126	606.316
		T/T	5 (2.26)	1 (0.50)	0.217 (0.011–1.362)	0.165		
	overdominant	C/C + T/T	182 (82.35)	174 (86.14)	1	–	591.689	607.879
		C/T	39 (17.65)	28 (13.86)	0.764 (0.446–1.295)	0.320		
	log-additive	–	–	–	0.667 (0.413–1.057)	0.090	589.722	609.912
LATS2 rs9509492	codominant	A/A	87 (39.01)	74 (36.82)	1	–	595.106	615.355
		A/G	98 (43.95)	96 (47.76)	1.147 (0.754–1.746)	0.522		
		G/G	38 (17.04)	31 (15.42)	0.964 (0.544–1.699)	0.898		
	dominant	A/A	87 (39.01)	74 (36.82)	1	–	593.488	609.687
		A/G + G/G	136 (60.99)	127 (63.18)	1.096 (0.739–1.627)	0.649		
	recessive	A/A + A/G	185 (82.96)	170 (84.58)	1	–	593.516	609.715
		G/G	38 (17.04)	31 (15.42)	0.894 (0.529–1.500)	0.672		
	overdominant	A/A + G/G	125 (56.05)	105 (52.24)	1	–	593.123	609.322
		A/G	98 (43.95)	96 (47.76)	1.160 (0.790–1.702)	0.449		
	log-additive	–	–	–	1.013 (0.772–1.329)	0.927	593.687	609.886

^a^adjusted for gender and age

### Association between *LATS1* and *LATS2* SNPs and risk of non-cardia GC

Results showed that *LATS1* rs9393175 was associated with the risk of non-cardia GC in codominant, dominant, and overdominant models. The overdominant genetic model was more suitable with the lowest AIC and BIC. *LATS2* rs9509492 was associated with the risk of non-cardia GC in the codominant, dominant, recessive, and log-additive models. The log-additive genetic model was more suitable with the lowest AIC and BIC. No associations were found between other SNPs and non-cardia GC (Table 4). Furthermore, no haploid blocks were formed between the four SNPs of *LATS2*.

**Table 4:** Association between 5 SNPs and risk of non-cardia gastric cancer under different genetic models

Locus	Model	Genotype	Control [n(%)]	Case [n(%)]	OR (95%CI)^a^	*P*	AIC	BIC
LATS1 rs9393175	codominant	G/G	253 (59.53)	253 (67.29)	1	–	1092.330	1115.760
		A/G	149 (35.06)	101 (26.86)	0.696 (0.509–0.949)	0.022		
		A/A	23 (5.41)	22 (11.26)	0.906 (0.486–1.683)	0.753		
	dominant	G/G	253 (59.53)	253 (67.29)	1	–	1090.969	1109.713
		A/A + A/G	172 (40.47)	123 (32.71)	0.725 (0.540–0.972)	0.032		
	recessive	G/G + A/G	402 (94.59)	354 (94.15)	1	–	1095.593	1114.337
		A/A	23 (5.41)	22 (5.85)	1.018 (0.551–1.877)	0.954		
	overdominant	G/G + A/A	276 (64.94)	275 (73.14)	1	–	1090.429	1109.173
		A/G	149 (35.06)	101 (26.86)	0.702 (0.516–0.952)	0.024		
	log-additive	–	–	–	0.814 (0.641–1.030)	0.088	1092.663	1111.406
LATS2 rs558614	codominant	T/T	123 (29.85)	90 (23.81)	1	–	1081.499	1104.859
		C/T	197 (47.82)	192 (50.79)	1.287 (0.916–1.812)	0.147		
		C/C	92 (22.33)	96 (25.40)	1.375 (0.923–2.052)	0.118		
	dominant	T/T	123 (29.85)	90 (23.81)	1	–	1079.635	1098.323
		C/C + C/T	289 (70.15)	288 (76.19)	1.315 (0.955–1.815)	0.094		
	recessive	T/T + C/T	320 (77.67)	282 (74.60)	1	–	1081.613	1100.301
		C/C	92 (22.33)	96 (25.40)	1.168 (0.838–1.628)	0.360		
	overdominant	T/T + C/C	215 (52.18)	186 (49.21)	–	–	1081.954	1100.642
		C/T	197 (47.82)	192 (50.79)	1.107 (0.834–1.470)	0.480		
	log-additive	–	–	–	1.176 (0.964–1.436)	0.111	1079.912	1098.600
LATS2 rs9552315	codominant	T/T	105 (24.82)	100 (26.46)	1	–	1096.314	1119.743
		C/T	207 (48.94)	195 (51.59)	1.034 (0.735–1.456)	0.848		
		C/C	111 (26.24)	83 (21.96)	0.812 (0.544–1.212)	0.309		
	dominant	T/T	105 (24.82)	100 (26.46)	1	–	1096.153	1114.896
		C/C + C/T	318 (75.18)	278 (73.54)	0.956 (0.693–1.321)	0.785		
	recessive	T/T + C/T	312 (73.76)	295 (78.04)	1	–	1094.350	1113.094
		C/C	111 (26.24)	83 (21.96)	0.795 (0.570–1.104)	0.172		
	overdominant	T/T + C/C	216 (51.06)	183 (48.41)	1	–	1095.351	1114.095
		C/T	207 (48.94)	195 (51.59)	1.144 (0.863–1.516)	0.350		
	log-additive	–	–	–	0.903 (0.740–1.102)	0.317	1095.226	1113.970
LATS2 rs7317471	codominant	C/C	350 (82.74)	302 (79.89)	1	–	1097.992	1121.421
		C/T	67 (15.84)	69 (18.25)	1.188 (0.816–1.729)	0.368		
		T/T	6 (1.42)	7 (1.85)	1.386 (0.449–4.402)	0.566		
	dominant	C/C	350 (82.74)	302 (79.89)	1	–	1096.061	1114.804
		C/T + T/T	73 (17.26)	76 (20.11)	1.204 (0.839–1.729)	0.314		
	recessive	C/C + C/T	417 (98.58)	371 (98.15)	1	–	1096.804	1115.547
		T/T	6 (1.42)	7 (1.85)	1.345 (0.437–4.264)	0.602		
	overdominant	C/C + T/T	356 (84.17)	309 (81.75)	1	–	1096.322	1115.065
		C/T	67 (15.84)	69 (18.25)	1.180 (0.812–1.717)	0.385		
	log-additive	–	–	–	1.185 (0.861–1.633)	0.298	1095.993	1114.736
LATS2 rs9509492	codominant	A/A	161 (37.97)	180 (47.24)	1	–	1094.648	1118.102
		A/G	194 (45.75)	161 (42.26)	0.747 (0.552–1.010)	0.058		
		G/G	69 (16.27)	40 (10.50)	0.525 (0.333–0.818)	0.005		
	dominant	A/A	161 (37.97)	180 (47.24)	1	–	1095.087	1113.850
		A/G + G/G	263 (62.03)	201 (52.76)	0.688 (0.518–0.915)	0.010		
	recessive	A/A + A/G	355 (83.73)	341 (89.50)	1	–	1096.241	1115.004
		G/G	69 (16.27)	40 (10.50)	0.609 (0.397–0.923)	0.021		
	overdominant	A/A + G/G	230 (54.25)	220 (57.74)	1	–	1100.815	1119.578
		A/G	194 (45.75)	161 (42.26)	0.871 (0.657–1.156)	0.339		
	log-additive	–	–	–	0.731 (0.594–0.897)	0.003	1092.686	1111.449

^a^adjusted for gender and age

### Association between five SNPs and expression of LATS1 and LATS2 proteins in non-cardia GC tissues

We evaluated the expression of LATS1 and LATS2 proteins by immunohistochemistry among the non-cardia GC tissues and normal tissues adjacent to cancer in our pre-study [14]. The results showed that the expression of LATS1 and LATS2 in non-cardia GC tissue was significantly lower than that in normal gastric tissue adjacent to cancer. We analyzed the association between five SNPs and expression of LATS1 or LATS2 proteins in 111 non-cardia GC tissue samples by the logistic regression method in five different genetic models. The results showed that no associations were found (Table 5). Furthermore, no haploid blocks were formed between the four SNPs of *LATS2*.

**Table 5:** Association between 5 SNPs and expression of LATS1 and LATS2 under different genetic models

Locus	Model	Genotype	Positive expression N(%)	Negative expression N(%)	OR (95% CI)^a^	*P*	AIC	BIC
LATS1 rs9393175	codominant	G/G	30 (62.50)	40 (65.57)	1	–	157.896	171.353
		A/G	15 (31.25)	16 (26.23)	1.208 (0.508–2.869)	0.667		
		A/A	3 (6.25%)	5 (8.20)	0.795 (0.152–3.535)	0.767		
	dominant	G/G	30 (62.50)	40 (65.57)	1	–	156.162	166.927
		A/A + A/G	18 (37.50)	21 (34.43)	1.110 (0.497–2.467)	0.798		
	recessive	G/G + A/G	45 (93.75)	56 (91.80)	1	–	156.082	166.847
		A/A	3 (6.25)	5 (8.20)	0.750 (0.146–3.258)	0.706		
	overdominant	G/G + A/A	33 (68.75)	45 (73.77)	1	–	155.985	166.751
		A/G	15 (31.25)	16 (26.23)	1.237 (0.527–2.897)	0.623		
	log-additive	–	–	–	1.012 (0.544–1.863)	0.970	156.225	166.991
LATS2 rs558614	codominant	T/T	20 (26.67)	6 (17.14)	1	–	144.846	158.348
		C/T	35 (46.67)	21 (60.00)	0.505 (0.163–1.407)	0.208		
		C/C	20 (26.67)	8 (22.86)	0.739 (0.208–2.522)	0.630		
	dominant	T/T	20 (26.67)	6 (17.14)	1	–	143.431	154.233
		T/C + C/C	55 (73.33)	29 (82.86)	0.570 (0.190–1.513)	0.280		
	recessive	T/T + C/T	55 (73.33)	27 (77.14)	1	–	144.519	155.321
		C/C	20 (26.67)	8 (22.86)	1.200 (0.478–3.229)	0.705		
	overdominant	C/C + T/T	40 (53.33)	14 (40.00)	1	–	143.079	153.881
		C/T	35 (46.67)	21 (60.00)	0.593 (0.258–1.336)	0.211		
	log-additive	–	–	–	0.882 (0.491–1.572)	0.670	144.483	155.285
LATS2 rs9552315	codominant	T/T	25 (33.33)	7 (20.59)	1	–	141.158	154.615
		C/T	31 (41.33)	20 (58.82)	0.409 (0.139–1.100)	0.087		
		C/C	19 (25.33)	7 (20.59)	0.716 (0.207–2.451)	0.591		
	dominant	T/T	25 (33.33)	7 (20.59)	1	–	140.308	151.073
		T/C + C/C	50 (66.67)	27 (79.41)	0.489 (0.174–1.244)	0.149		
	recessive	T/T + C/T	56 (74.67)	27 (79.41)	1	–	142.279	153.044
		C/C	19 (25.33)	7 (20.59)	1.283 (0.493–3.632)	0.621		
	overdominant	C/C + T/T	44 (58.67)	14 (41.18)	1	–	139.447	150.213
		C/T	31 (41.33)	20 (58.82)	0.478 (0.205–1.089)	0.082		
	log-additive	–	–	–	0.836 (0.473–1.468)	0.533	142.140	152.905
LATS2 rs7317471		C/C	63 (85.14)	26 (74.29)	1	–	142.226	152.992
		C/T	11 (14.86)	9 (25.71)	0.509 (0.186–1.413)	0.187		
LATS2 rs9509492	codominant	A/A	34 (45.33)	16 (44.44)	1	–	147.894	161.441
		A/G	32 (42.67)	18 (50.00)	0.842 (0.364–1.936)	0.686		
		G/G	9 (12.00)	2 (5.56)	2.138 (0.478–15.146)	0.366		
	dominant	A/A	34 (45.33)	16 (44.44)	1	–	147.305	158.143
		A/G + G/G	41 (54.67)	20 (55.56)	0.972 (0.433–2.167)	0.945		
	recessive	A/A + A/G	66 (88.00)	34 (94.44)		–	146.058	156.896
		G/G	9 (12.00)	2 (5.56)	2.332 (0.559–15.911)	0.297		
	overdominant	A/A + G/G	43 (57.33)	18 (50.00)	1	–	146.805	157.644
		A/G	32 (42.67)	18 (50.00)	0.748 (0.334–1.670)	0.478		
	log-additive	–	–	–	1.147 (0.623–2.160)	0.662	147.118	157.956

^a^adjusted for gender and age

## Discussion

GC is one of the most common malignant tumors with a large number of new cases and deaths every year globally. Asia is a region with a high incidence of GC, accounting for about half of the total number of cases worldwide, while China is one of the countries with a high incidence of GC in Asia. Although the incidence and mortality of GC have been decreasing in recent years, they are still at the forefront of all malignant tumors. According to its anatomical location, GC can be divided into cardia and non-cardia, which have big differences in the mechanism, carcinogenesis, clinical manifestations, treatment, and prognosis. Cardia GC is similar to esophageal cancer regarding clinical characteristics, etiology, and pathology and epidemiology. Therefore, this experiment studied non-cardia GC to avoid sample clinical heterogeneity that could affect the results of the study.

The Hippo signaling pathway is a tumor inhibition pathway that was discovered in recent years, of which LATS1 and LATS2 kinase are two important components and have many important biological functions. LATS1 and LATS2 are involved in the occurrence and development of GC [15].

*H. pylori* infection is an important factor causing GC. Genome-wide scanning and case-control studies have confirmed that *H. pylori* infection is associated with individual genetic polymorphism [7]. In this study, we found that rs9552315 of *LATS2* gene was associated with *H. pylori* infection, and the dominant model was most suitable with the lowest AIC and BIC. Compared with the TT genotype, the CC + CT genotype can reduce the risk of *H. pylori* infection. The other four SNPs were not associated with *H. pylori* infection. At present, no correlation analysis between these five SNPs and *H. pylori* infection has been reported, so our results need to be further verified.

Among the five selected SNPs, rs7317471 and rs9509492 were used to study the mortality of hepatocellular carcinoma (HCC) [16]. The results showed that rs7317471 was associated with mortality from HCC, and rs9509492 was considered to be an independent prognostic indicator of overall survival rate of HCC patients, while rs9509492 was not associated with the mortality of HCC. Moreover, Sebio [17] studied the association between rs558614 and rs9552315 and colorectal cancer, and the results showed that the two SNPs were not associated with the incidence risk of colorectal cancer. In this study, we analyzed the association between five SNPs of *LATS1* and *LATS2* genes and the risk of non-cardia GC. The results showed that *LATS1* rs9393175 was associated with the risk of non-cardia GC in an overdominant model which was most suitable with the lowest AIC and BIC. Compared with the GG + AA genotype, carrying the AG genotype may reduce the risk of non-cardia GC. Therefore, the AG genotype may be a protective factor against non-cardia GC in the Baotou Han population. Besides, *LATS2* rs9509492 was found to be associated with the risk of non-cardia GC. The log-additive genetic model is most suitable with the lowest AIC and BIC. Furthermore, no associations were found between *LATS2* SNPs rs558614, rs9552315, rs7317471 and risk of non-cardia GC. To the best of our knowledge, this is the first report of associations between these five SNPs and non-cardia GC, so our experimental results need further confirmation.

Furthermore, the relationship between polymorphisms in the *LATS1* and *LATS2* genes and their protein expression levels were analyzed. However, no correlations were found between these five SNPs and their protein expression levels, suggesting that gene mutations at five loci of the two genes may not affect their protein expression levels. It is also possible that these five SNPs had small effects on protein expression levels that were not detected due to the small sample size of only 111 non-cardia GC tissue samples. Therefore, our experimental results need to be further confirmed by expanding the sample size.

There are several limitations to our study. Firstly, we recruited non-cardia gastric cancer cases from one hospital and selected normal controls from another two hospitals, which might not be representative of the general population and resulted in potential selection bias. Secondly, although the infection of *H. pylori* was correlated with non-cardia gastric cancer risk, it is difficult to measure *H. pylori* infection in gastric cancer patients. Gastric cancer is a multigenic disease, and patients generally develop into gastric cancer through chronic atrophic gastritis, intestinal metaplasia, low-level neoplasia, high-level neoplasia stages. *H. pylori* lose its colonized soil in atrophic body gastritis and disappear slowly during gastric carcinogenesis [18, 19]. Moreover, in a long disease progression, most patients were treated with the antibiotic, which resulted in the loss of *H. pylori*. Lack of available information on *H. pylori* infection status in patients with non-cardia gastric cancer limited us to adjust the potential confounding bias of this risk factor.

## Conclusions

In this experiment, our group studied the association between these five SNPs and *H. pylori* infection and explored the association between *LATS1* and *LATS2* SNPs and non-cardiac GC for the first time, to the best of our knowledge. The results of this study indicated that some SNPs of the *LATS1* and *LATS2* genes were involved in the pathogenesis of *H. pylori* infection and non-cardia GC, of which *LATS2* rs9552315 was associated with *H. pylori* infection. Besides, compared with the TT genotype, the CC + CT genotype appeared to reduce the risk of *H. pylori* infection and may be a protective factor against *H. pylori* infection in the Baotou Han population. *LATS1* rs9393175 and *LATS2* rs9509492 were associated with the risk of non-cardia GC, and AG and GG genotypes may be protective factors against GC in the Baotou Han population. These two SNPs may become biomarkers for GC screening and provide a new approach for the targeted therapy of GC. However, non-cardia GC patients and normal control samples were from the Han population in Baotou, Inner Mongolia, so the results only represent the genetic characteristics of the Han population in this area, and the sample size was also low. In the future, other regions and other ethnic groups need to be studied, and the sample size should be expanded to further clarify the relationship between these five SNPs and *H. pylori* infection and non-cardia GC.

## Data Availability

The datasets generated and analyzed during the present study are available from the corresponding author on reasonable request. All data generated or analyzed during this study are included in this published article.

## Abbreviations

LATS1: Large tumor suppressor kinase 1
LATS2: Large tumor suppressor kinase 2
GC: Gastric cancer
SNP: Single nucleotide polymorphism
*H. pylori*: *Helicobacter pylori*
ELISA: Enzyme-linked immunosorbent assay
OR: Odds ratio
CI: Confidence interval
HWE: Hardy Weinberg Equilibrium
AIC: Akaike Information Criterion
BIC: Bayesian Information Criterion
HCC: Hepatocellular carcinoma
